# High-frequency rTMS over the left DLPFC improves the response inhibition control of young healthy participants: an ERP combined ^1^H-MRS study

**DOI:** 10.3389/fpsyg.2023.1144757

**Published:** 2023-05-18

**Authors:** Yanmin Li, Jianmin Pang, Jing Wang, Wei Wang, Qianlan Bo, Licun Lei, Xiayue Wang, Mingwei Wang

**Affiliations:** ^1^Department of Neurology, The First Hospital of Hebei Medical University, Shijiazhuang, Hebei, China; ^2^Brain Aging and Cognitive Neuroscience Laboratory of Hebei Province, Shijiazhuang, Hebei, China; ^3^Department of Neurology, Hebei Hospital of Xuanwu Hospital Capital Medical University, Shijiazhuang, Hebei, China; ^4^Department of Respiratory Medicine, Harrison International Peace Hospital, Hengshui, Hebei, China; ^5^Department of Radiology, The First Hospital of Hebei Medical University, Shijiazhuang, Hebei, China

**Keywords:** high-frequency, repetitive transcranial magnetic stimulation (rTMS), young healthy participants, response inhibition, event-related potentials (ERPs), magnetic resonance spectroscopy (^1^H-MRS)

## Abstract

**Introduction:**

Unlike the effect of repetitive transcranial magnetic stimulation (rTMS) in treating neuropsychiatric diseases, little is known about how personal factors might account for the disparity of results from studies of cognition and rTMS. In this study, we investigated the effects of high-frequency rTMS on response inhibition control and explored the time course changes in cognitive processing and brain metabolic mechanisms after rTMS using event-related potentials (ERPs) and magnetic resonance spectroscopy (^1^H-MRS).

**Methods:**

Participants were all right-handed and were naive to rTMS and the Go/NoGo task. Twenty-five healthy young participants underwent one 10 Hz rTMS session per day in which stimulation was applied over the left dorsolateral prefrontal cortex (DLPFC), and a homogeneous participant group of 25 individuals received a sham rTMS treatment for 1  week. A Go/NoGo task was performed, an electroencephalogram (EEG) was recorded, and ^1^H-MRS was performed.

**Results:**

The results revealed that there was a strong trend of decreasing commission errors of NoGo stimuli by high frequency rTMS over the left DLPFC, whereas there was no significant difference between before and after rTMS treatment with respect to these parameters in the sham rTMS group. High-frequency rTMS significantly increased the amplitude of NoGo-N2 but not Go-N2, Go-P3, or NoGo-P3. The myo-inositol /creatine complex (MI/Cr) ratio, indexing cerebral metabolism, in the left DLPFC was decreased in the rTMS treated group.

**Discussion:**

This observation supports the view that high-frequency rTMS over the left DLPFC has the strong tendency of reducing commission errors behaviorally, increase the amplitude of NoGo-N2 and improve the response inhibition control of healthy young participants. The results are consistent with the excitatory properties of high frequency rTMS. We suggest that the increase in the NoGo-N2 amplitude may be related to the increased excitability of the DLPFC-anterior cingulate cortex (ACC) neural loop. Metabolic changes in the DLPFC may be a possible mechanism for the improvement of the response inhibition control of rTMS.

## 1. Introduction

Repetitive transcranial magnetic stimulation (rTMS) has been widely used in both the neuropsychiatric and the neuroscience fields for ameliorating clinical symptoms of neuropsychiatric disorders (Jääskeläinen et al., 2019) as a noninvasive, well-tolerated tool and a modulator of brain function. Because of its special physical characteristics, rTMS techniques allow researchers to evaluate the modifications of brain electric activity induced by stimulation both in healthy and pathologic conditions. Studies have confirmed that rTMS can improve cognitive function, such as word recall, speech memory, associative memory, selective attention, speech expression and other executive functions, of patients with depression, cerebrovascular disease, and Alzheimer’s disease (Little et al., 2000; Sedlackova et al., 2008; Alcala Lozano et al., 2019; Wang et al., 2019; Asgharian Asl and Vaghef, 2022; Fitzgerald and Daskalakis, 2022). Guse et al., (2010) reviewed that rTMS at 10, 15 or 20 Hz, applied over the left DLPFC and an individual motor threshold of 80–110%, is most likely to cause significant cognitive improvement.

In recent years, an increasing number of researchers have been interested in the effect of rTMS on the cognitive function of healthy people, and many studies have achieved positive results. For example, Turriziani et al. found that rTMS over the right dorsolateral prefrontal cortex (DLPFC) enhanced recognition memory in 100 healthy participants aged 20–35 years (Turriziani et al., 2012). A similar improvement was observed by Hwang et al. (2010), who found that healthy young males showed fewer commission errors in a Conners’ continuous performance test after rTMS. Another study found that one session of high-frequency (10 Hz) rTMS over the left DLPFC improved the behavioral performance of a Stroop task instead of sham stimulation in healthy young females (Vanderhasselt et al., 2006). Consistent with these data, our previous research also showed that multiple sessions of high-frequency rTMS over the left DLPFC can decrease RTs under both congruent and incongruent conditions of the Stroop task, increase neural activity in the prefrontal areas by inducing an electrophysiologically excitatory effect and enhance the efficiency of resources to deploy for conflict resolution during multiple stages of cognitive control processing in healthy young people (Li et al., 2017). Researchers have demonstrated the ability of rTMS over the left DLPFC to enhance cognitive functioning, including visuospatial, aural, sustained attention, working memory and executive function, in healthy individuals (McKinley et al., 2012).

The inhibition control is considered to be a key aspect of executive function and is not yet uniformly and clearly defined. A generally accepted view that it is not a unitary function and encompasses at least two components: interference inhibition and motor inhibition (Bari and Robbins, 2013; Mirabella, 2021). The interference inhibition assesses the ability to resolve response conflict while motor response inhibition, which it is measured via the Go/NoGo task or the countermanding task, refers to the capacity to inhibit prepotent responses and suppress inappropriate actions (Tong et al., 2021). Furthermore, motor inhibition has two neuropsychological sub-domains: reactive inhibition, i.e., the ability to stop a response immediately when a stop instruction is presented, and proactive inhibition, i.e., the ability to adapt the motor strategy according to the context where an individual is embedded. The present study will focus on reactive response inhibition. It plays a crucial role in making correct behavior decisions to adapt to the requirements of task changes (Liu and Zhang, 2020). A large and complex neural network is involved in the neural processing of response inhibition, encompassing both frontal and parietal areas as well as subcortical brain structures, e.g., the basal ganglia, supplementary motor area (SMA), pre-supplementary motor area (pre-SMA), inferior frontal gyrus (IFG), dorsolateral prefrontal cortex (DLPFC), anterior cingulate cortex (ACC), frontal pole cortex (FPC) et al. (Mirabella, 2014). Among these numerous brain structures, the DLPFC-ACC loop has garnered much attention. The previous functional neuroimaging studies have suggested that inhibition is implemented by a right-lateralized network centered around the right inferior frontal gyrus (Simmonds et al., 2008; Gavazzi et al., 2021; Aron et al., 2003; Aron et al., 2004; Aron et al., 2014). However, support for this position has not been consistent. Other data indicate that the left hemisphere also plays a critical role in inhibitory control (Swick et al., 2008; Gavazzi et al., 2019). In the present study, the role of the left DLPFC-ACC loop in response inhibition control will be discussed. From an electrophysiological standpoint, N2 and P3 in response to NoGo stimuli are two important components for the evaluation of response inhibition control that are measured by event-related potential (ERP) technique.

The N2 component, the second negative peak in the averaged ERP waveform, peaks between 200 ms and 350 ms after stimulus onset with an anterior scalp distribution and is typically larger for NoGo stimuli than Go stimuli in a Go/NoGo task. According to the literature, NoGo-N2 reflects an earlier step of the response inhibition control process, i.e., the monitoring or detection of response conflict between the internal representation of the Go response and the NoGo stimulus (Kok, 1986; Smith et al., 2008; Wu et al., 2010; Megías et al., 2017). P3 is another important ERP component that is characterized by a larger positive wave that peaks within the range of 300–500 ms and is larger for NoGo stimuli than Go stimuli in a Go/NoGo task like the N2 component. NoGo-P3 reflects a later stage of the response inhibitory process, which represents the underpinning of the action outcome assessment (Bruin et al., 2001; Smith et al., 2008). ACC is involved in the generation of the two above described ERP components of response inhibition control (Munro et al., 2007).

Deficits in response inhibition control are related to many diseases, such as attention deficit hyperactivity disorder (ADHD) (Smith et al., 2004; van Hulst et al., 2018; Suarez et al., 2021), posttraumatic stress disorder (PTSD) (Shucard et al., 2008), depression (Kaiser et al., 2003), obsessive-compulsive disorder (OCD) and Tourette syndrome (TS) (Mancini et al., 2018), primary motor stereotypies (Mirabella et al., 2020), autism spectrum disorders (ASD) (Schmitt et al., 2018), and Parkinson’s disease (PD) (Bokura et al., 2005; Mirabella et al., 2017; di Caprio et al., 2020). The amplitudes of NoGo-N2 and NoGo-P3 were also significantly smaller in the PD group than in the control group (Bokura et al., 2005). In terms of latency, another study found that the latency of NoGo-N2 and NoGo-P3 in young people with PTSD was shorter than that of the healthy control group (Wu et al., 2010). In a stop-signal task, the stop-signal response time (SSRT) was longer and the P3 amplitudes were smaller in the depressed patients than the healthy controls. rTMS improved the depression symptoms significantly, reduced SSRT, and increased the P3 amplitude in patients with depression. It indicated that rTMS might represent an effective approach to promotes response inhibition control in patients with major depressive disorder (Yu et al., 2022).

However, the effect of rTMS on response inhibition control in healthy participants remains inconsistent. Grosshenrich (Grossheinrich et al., 2013) obtained a positive result that 1 Hz rTMS over the left DLPFC can affect the amplitude of N2 in a Go/NoGo task, while rTMS over the medial prefrontal cortex (mPFC) has a trend of increasing the amplitude of P3. However, two other rTMS studies focused on its effects on response inhibition control achieved negative results. Upton et al. (2010) found that low frequency (1 Hz) rTMS stimulation over the left and right DLPFC did not enhance behavioral data and ERP waveforms of response inhibition control in a stop-signal task. Huang et al. (2004) also found that 5 Hz short-term rTMS over the left DLPFC will not enhance or deteriorate cognition in a Go/NoGo task with a crossover design. These negative results may be due to the rTMS parameters used in these studies, such as stimulation type, location, frequency, intensity, treatment duration and experimental design method. In a previous study, it has been shown that longer stimulation trains would induce more lasting effects (Eisenegger et al., 2008). In other words, more pulses, higher intensity and longer treatment durations mean better effects to some extent with the control of safety and adverse effects of rTMS. Meanwhile, it remains unclear which stages of the time course of the neural processing of response inhibition control are affected and how these stages are altered by rTMS.

The mechanisms of action of rTMS are complicated and still largely unclear. One of the putative mechanisms was linked to a change of activity in the DLPFC and the ACC (de Raedt et al., 2010; van den Heuvel et al., 2013; Esslinger et al., 2014). Our previous research (Li et al., 2017) found that high frequency rTMS can decrease reaction time (RTs) under both congruent and incongruent conditions and increase the amplitudes of both N2 and N450 compared with sham rTMS in a Stroop task with ERP technique. ERPs are useful for the examination of cognitive processing variations because this method provides millisecond-level temporal resolution of neural activity. ERPs have been applied in many rTMS-related studies and have aided the investigation of rTMS effects on the time course of cognitive processing. Moreover, changes in cerebral metabolism, for example, increased glucose consumption, and glutamatergic neurotransmission after rTMS stimulation not only under the coil but also in remote brain areas (Speer et al., 2000; Strafella et al., 2001) with proton magnetic resonance spectroscopy (^1^H-MRS) technology are involved. ^1^H-MRS provides a unique opportunity to assess brain metabolism levels *in vivo*. At present, ^1^H-MRS has been widely used in clinical and scientific research. For example, ^1^H-MRS is used in patients with mild cognitive impairment, Alzheimer’s disease and elderly people with normal cognition to evaluate changes in brain metabolism (Schuff et al., 1999; Kantarci et al., 2013; Kandimalla et al., 2018). However, few studies have used ERPs and ^1^H-MRS to explore the mechanism of rTMS.

The aims of the present study were as follows: (1) to determine whether high-frequency rTMS over the left DLPFC can improve the response inhibition control of healthy people from a behavioral point of view, (2) to clarify the intrinsic electrophysiological mechanism of rTMS on response inhibition control using ERP technique and to provide a possible and theoretical basis for rTMS on response inhibition control in healthy subjects, and (3) to study the effect of rTMS on local brain metabolism using ^1^H-MRS. High frequency rTMS (10 Hz) was employed for one session a day for seven consecutive days, and a Go/NoGo task was utilized before and after the rTMS treatment. Based on the excitatory effect of high-frequency rTMS, we expected the behavioral performance of the Go/NoGo task to be improved, the amplitudes of NoGo-N2 to be larger and NoGo-P3 to be smaller in the rTMS group than in the sham rTMS group, and the brain metabolism of the local area was changed.

## 2. Participants and methods

### 2.1. Experimental design

**Figure 1:**
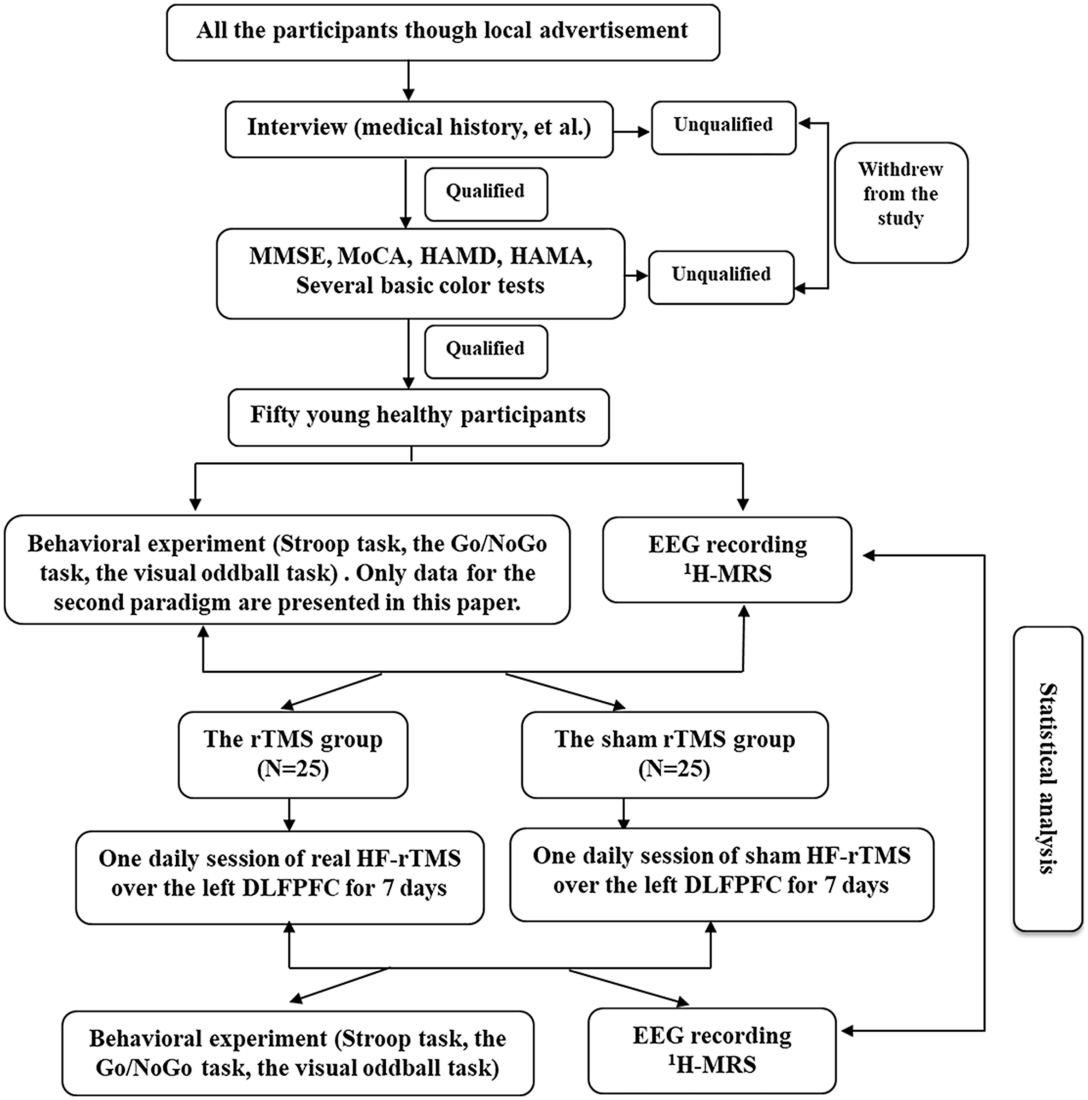
The experimental design flow chart of the study.

We used a single-blind, randomized, sham-controlled design to examine the effects of high frequency rTMS. Participants were blinded to the sequence of the rTMS protocols until the end of the study. The experimental design flow chart of the study is shown in Figure 1.

### 2.2. Participants

Fifty healthy young postgraduates with an age range of 24–30 years (mean 26.8 ± 1.43 years) at Hebei Medical University in China were involved in the present study. Twenty-six of the participants were females, and 24 were males. All participants were screened as normal on mood and cognitive scales, e.g., scores on two mood scales [24-item Hamilton Depression Rating Scale (HAMD) (Hamilton, 1967) and Hamilton Anxiety Scale (HAMA) (Hamilton, 1959)] were each less than seven, and scores on two cognitive scales [Mini Mental State Examination (MMSE) (Folstein et al., 1975) and Montreal Cognitive Assessment (MoCA) [Nasreddine et al., 2005)] were each greater than 27 (see Questionnaires). None of the participants had any neurological, psychiatric or other medical history, for example, a history of epilepsy or head injury, neurologic or psychiatric disease or other health problems. They did not have any contraindications to rTMS (for example, cardiac pacemaker placement, metal implants in the head or neck region, fixation elements, tattoos on any part of the body, artificial joints, and medication use) or previous rTMS experience. All the participants were self-reported right-handed, without major life events during the study period, and had normal or corrected-to-normal vision and normal color vision as assessed by several basic color tests. Although self-reports are imprecise, previous study has been shown that handedness does not impact inhibitory proficiency in young healthy individuals (Mancini and Mirabella, 2021). All the participants were required to consume no alcohol throughout the procedure, and those who had alcohol use and major life events (based on self-report) during the study period were excluded.

All participants were randomly allocated to the rTMS group (25 participants, mean age 26.6 ± 1.15 years) or the sham rTMS group (25 participants, mean age 26.9 ± 1.68 years) with the limitation that age and gender composition were matched across the two groups. Each group had 12 males and 13 females. The group memberships of the participants (real rTMS or sham rTMS) were not revealed until the whole experiment was completed. We managed the data of participants by serial number, name, and age to prevent confusion.

The study was performed in accordance with ethical guidelines and received ethical approval from the Medical Ethics Committee of the First Hospital of Hebei Medical University. All participants provided written informed consent and were awarded ¥150 following completion of the whole experiment.

### 2.3. General procedures

The present study reports the results obtained from a larger study addressing the effect of a high frequency of rTMS on cognition. The initial data were obtained under three experimental paradigms: the classical Stroop color-word paradigm, the Go/NoGo paradigm, and the visual oddball paradigm. The general procedures used in this experiment have been described in detail in previous studies (Li et al., 2017). Only data for the second paradigm are presented in this paper.

First, qualified participants were recruited. At first, on arriving at the laboratory, participants had to carry out a series of questionnaires for evaluating mood states and cognitive integrity (described below) and several basic color tests whose main purpose was to eliminate color vision disorders and complete the Stroop task. Following successful completion of these qualifying data, the participants were seated relaxed on a comfortable armchair in a dimly lit and electrically isolated room and fitted with 64-scalp electrodes. The Go/NoGo task (see descriptions below) was performed, and the behavioral data, electroencephalogram (EEG) data and brain metabolism data were collected prior to rTMS or sham rTMS administration. All of the initial data were collected 24 h prior to the first administration of rTMS or sham rTMS. The participants received one session a day of real rTMS or sham rTMS for seven consecutive days. After 1 week, participants were immediately reevaluated using mood questionnaires, the Go/NoGo task, EEG and ^1^H-MRS technology.

### 2.4. Questionnaires

The 24-item -HAMD (Hamilton, 1967) and the HAMA (Hamilton, 1959) were used to evaluate the mood states of the participants as a means of excluding ineligible participants. Cognitive integrity was measured by the MMSE (Folstein et al., 1975) and MoCA (Nasreddine et al., 2005).

### 2.5. rTMS procedure

Both real and sham rTMS applied over the left DLPFC were delivered by means of a MagStim Super-Rapid magnetic stimulator using a figure-eight-shaped coil (70 mm in diameter). This induces a maximum electrical field that peaks under the intersection of the two windings (Ren et al., 1995). The left DLPFC stimulation site was defined using the method of Pascual-Leone, which located the stimulation site 5 cm anterior to the area of the optimal primary motor cortex of the left hemisphere. This method has been reported to be accurate in targeting the DLPFC area (Haghighi et al., 2015). The motor threshold (MT) was determined individually before real and sham stimulation. The rTMS parameters were as follows: the stimulation intensity was 110% of the motor threshold of the right abductor pollicis brevis muscle, and the stimulation frequency was 10 Hz. Each rTMS protocol contained 1,350 pulses. Forty-five trains of 3 s duration were divided into 3 blocks, and the intertrain interval was 10 s in each block; the interblock interval was 20 s. It takes approximately 10.5 min per day. Real and sham rTMS were performed at the same location on the skull; however, for sham stimulation, the figure-eight-shaped coil was held at a 90° angle, only resting on the scalp with one edge. Each individual received stimulation at the same time of day for all sessions (sessions were performed from either 8:00–9:00 AM or 14:00–15:00 PM).

### 2.6. Stimuli

Stimuli were shown in the middle of a 17-inch computer screen with a black background. The white letter (“O” or “X”) was presented in the center of the screen with a visual angle of approximately 2.5° vertically and 2.2° horizontally that was viewed from a distance of approximately 70 cm.

### 2.7. Go/NoGo task

The stimuli consisted of Go and NoGo stimuli (probability of 20% NoGo and 80% Go trials). The participants were required to respond upon the appearance of the Go stimuli, e.g., the letter “X,” by pressing a button on the keyboard with the index finger of their dominant hand as quickly as possible and to refrain from responding upon the appearance of the NoGo stimuli (stop), e.g., the letter “O.” The consecutive presentation of two NoGo stimuli was avoided. Before the formal task, the participants completed one practice block, which consisted of 20 trials similar to those used in the experimental blocks. The experimental Go/NoGo task included two blocks that each consisted of 240 trials (for a total of 480 trials). The stimuli were presented for 150 ms with a random interstimulus interval of 1,200–1,500 ms. The participants completed the entire Go/NoGo task with 2 to 3 min breaks between two blocks. The association between the stimuli and Go/NoGo responses was counterbalanced across participants. The Go/NoGo task was programmed using E-Prime software, and the response time of Go trials, response accuracy for the Go condition and commission errors for NoGo trails were recorded.

### 2.8. EEG, ERP recording and data processing

During the Go/NoGo task, an EEG was recorded at 64 scalp sites according to the 10–20 international placement system with Ag/AgCl electrodes mounted in an elastic cap and NeuroScan 4.5 EEG/ERP recording system (amplifier type: SynAmps 2), with the reference on the left mastoids. For monitoring eye movements and blinks, the horizontal and vertical electrooculogram (EOG) was recorded with another four electrodes: two electrodes were placed above and below the left eye, and two electrodes were placed 1 cm next to the external canthus. The sampling rate was 1,000 Hz, an online bandpass filter of 0.01–200 Hz was used. The impedance of all electrodes was maintained below 5 kΩ.

EEG data were processed offline with NeuroScan 4.5 software. EEG data of all 64 electrodes were rereferenced to the average of the left and right mastoids. Ocular artifacts (blinks and eye movements) were removed from the EEG signal using a regression procedure implemented in Neuroscan 4.5 software (Semlitsch et al., 1986). Epochs with artifacts exceeding ±100 μV were excluded from further analysis. The averaged epochs for ERP were 1,000 ms, including 200 ms prestimuli as the baseline and 800 ms poststimuli. The trials with correct responses were averaged in each condition.

The mean amplitudes of N2 and P3 were calculated at the negative maximum between 200 and 330 ms and at the positive maximum between 330 and 400 ms, respectively. Fz, FCz, and Cz electrode points were chosen for statistical analysis. These time windows and sites were chosen consistent with the previous literature (Wu et al., 2010). We overlapped and averaged the stimulus-locked waveform of each correct response for each participant. Grand averaged ERPs were obtained by averaging all the waveforms of all participants. And the visual inspection of the grand-average waveforms conducted to determine where and when ERPs were maximal.

### 2.9. Magnetic resonance spectroscopy (^1^H-MRS)

A double gradient superconducting magnetic resonance system (1.5 T, GE Company, The United States) was used in the present study. The volume of interest (VOI) in the bilateral DLPFCwas selected by using the T2WI axial position. The volumeixel size was 15 mm × 15 mm × 15 mm. Point Resolution Spectral Sequence (PRESS) was used to obtain the spectrum. TR/TE = 2000/136 ms. Receive/transmit gain adjustment, voxel shimming, water suppression, and water scarcity scanning are performed by the automatic scanning procedure, and the baseline correction and phase correction were performed after obtaining the spectrum. The same VOI was selected at the symmetrical part of the other DLFPC on the T2WI axis image, and the spectrum was obtained using the same method. Typical compounds that can be measured in ^1^H-MRS include N-acetyl aspartate (NAA), creatine complex (Cr), choline-containing compound (Cho), and myo-inositol (MI). NAA is a neuronal compound exclusively found in mature neurons, and therefore is thought to be a marker of neuronal integrity and viability. The decrease of NAA concentration means the loss of neurons or the loss of neuronal function. Cr is the buffer of energy metabolism. Generally, the concentration of Cr is very stable under various pathological conditions. Cho is the precursor of phosphatidylcholine and acetylcholine, which exists in cholinergic neurons and has a higher content in glial cells than neurons. MI is the precursor of phosphatidylinositol and 4,5-diphosphate phosphatidylinositol, which can participate in intracellular information transmission. Since the concentration of Cr is basically constant, Cr is used as the reference for each metabolites, and the ratio of NAA/Cr, Cho/Cr and MI/Cr, can represent the concentration of metabolites in the brain.

**Figure 2:**
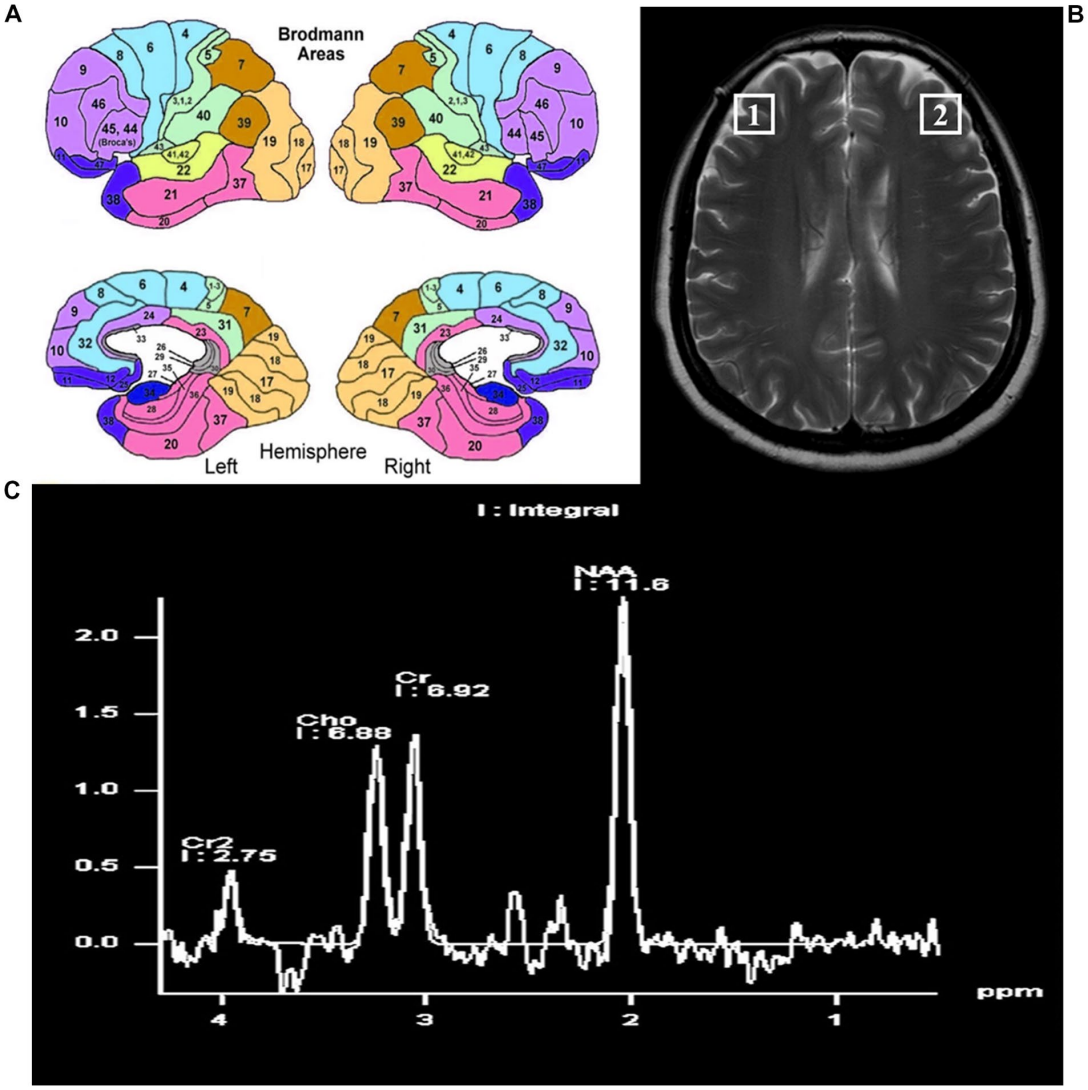
The locations of the bilateral dorsal lateral frontal cortex. **(A)** Pattern diagram of brain regions by Brodmann. **(B)** The locations of the bilateral dorsolateral prefrontal cortex on magnetic resonance. **(C)** The position and content of each metabolite by magnetic resonance spectroscopy.

The localization of the bilateral DLPFC refers to the Brodmann 46 area (BA46). In humans, BA46 roughly corresponds to the dorsolateral prefrontal lobe and part of Brodmann zone 9 (BA9), which is adjacent to BA46. The localization of the bilateral DLPFC is shown in Figure 2.

### 2.10. Data analysis

We adopted repeated-measures analyses of variance (ANOVA) with group (two levels: rTMS group and sham rTMS group) × time (two levels: T1, the baseline, before stimulation; T2, immediately after 7 days of rTMS or sham rTMS) × type of stimulus (two levels: Go stimulus, NoGo stimulus) (2 × 2 × 2) factors for response times (RTs) of Go trials, response accuracy of Go trials and commission errors of NoGo trials. As far as the psychometric scales are concerned, we used a two-way ANOVA with group (two levels: rTMS group and sham rTMS group) × time (two levels: T1, the baseline, before stimulation; T2, immediately after 7 days of rTMS or sham rTMS). Only the main effect of group, time, and type as well as interactions related to group were reported because we focused on the effect of rTMS compared with sham rTMS.

To analyze the mean amplitudes of N2 and P3 components, the ANOVA factors were group (two levels: rTMS group and sham rTMS group), time (two levels: T1, the baseline, before stimulation; T2, immediately after 7 days of rTMS or sham rTMS), type of stimulus (two levels: Go stimulus, NoGo stimulus), and electrode sites (Fz, FCz, and Cz) (2 × 2 × 2 × 3).

## 3. Results

### 3.1. Behavioral data of Go/NoGo task

There was a strong trend of decreasing commission errors of NoGo stimuli by high frequency rTMS over the left DLPFC, while the accuracy and RTs of Go stimuli were not affected.

#### 3.1.1. The RTs of correct Go trials in the Go/NoGo task

The RTs of correct Go trials data are presented in Table 1. For the RT analysis, no main group effect (*F*(1, 48) = 0.058, *p* = 0.812) or time effect (*F*(1, 48) = 0.611, *p* = 0.441) was observed. The interaction between time and group was not significant (*F*(1, 48) = 1.860, *p* = 0.183).

**Table 1:** The response accuracy for the Go condition and the commission errors for the NoGo condition in Go/NoGo task at two different time points in two groups.

Groups	The response accuracy for the Go condition		Commission errors for NoGo condition	
	T1	T2	T1	T2
rTMS group	99.8 ± 0.178%	99.7 ± 0.723%	12.17 ± 7.284%	9.64 ± 6.852%*
Sham rTMS group	99.7 ± 0.518%	99.7 ± 0.461%	11.00 ± 8.255%	12.37 ± 9.355%

T1, before stimulation; T2, immediately after 7 days of rTMS or sham rTMS; *p < 0.05.

#### 3.1.2. The response accuracy for the Go condition

For the response accuracy in the Go condition analysis, there were no main effects of group (*F*(1, 48) = 0.000, *p* = 1.000) or time (*F*(1, 48) = 0.481, *p* = 0.493). Additionally, no group × time interaction (*F*(1, 48) = 1.560, *p* = 0.221) was found.

#### 3.1.3. The commission errors in the NoGo condition

The commission errors data in the NoGo condition are presented in Table 1. For the commission errors in the NoGo condition analysis, no significant main effects for time (*F*(1, 48) = 0.254 *p* = 0.618) or group (*F*(1, 48) = 0.092, *p* = 0.764) were found. The *p* value of group × time interaction effect was 0.10 (*F*(1, 48) = 2.819). The result suggests that there is a trend of difference between the two groups at different time points. To further test this account, the Bayes factor was calculated with the Bayesian statistical theory. The analysis identified a Bayes factor (BF_10(group × time)_) of 4.86, which provides moderate evidence for group × time interaction. Further simple effect analysis revealed that the commission errors in the NoGo condition at T2 was significantly lower than that at T1 (*t* = 2.438, *p* = 0.028) in the rTMS group, whereas there was no significant difference between T1 and T2 with respect to these parameters in the sham rTMS group (*t* = −0.657, *p* = 0.521).

### 3.2. ERP data

The amplitude of NoGo-N2 is larger than Go-N2 in a Go/NoGo task. Multiple sessions of high-frequency rTMS over the left DLPFC increased NoGo-N2, while there were no effects on the amplitudes of Go-N2, Go-P3, and NoGo-P3.

#### 3.2.1. N2 component

**Figure 3:**
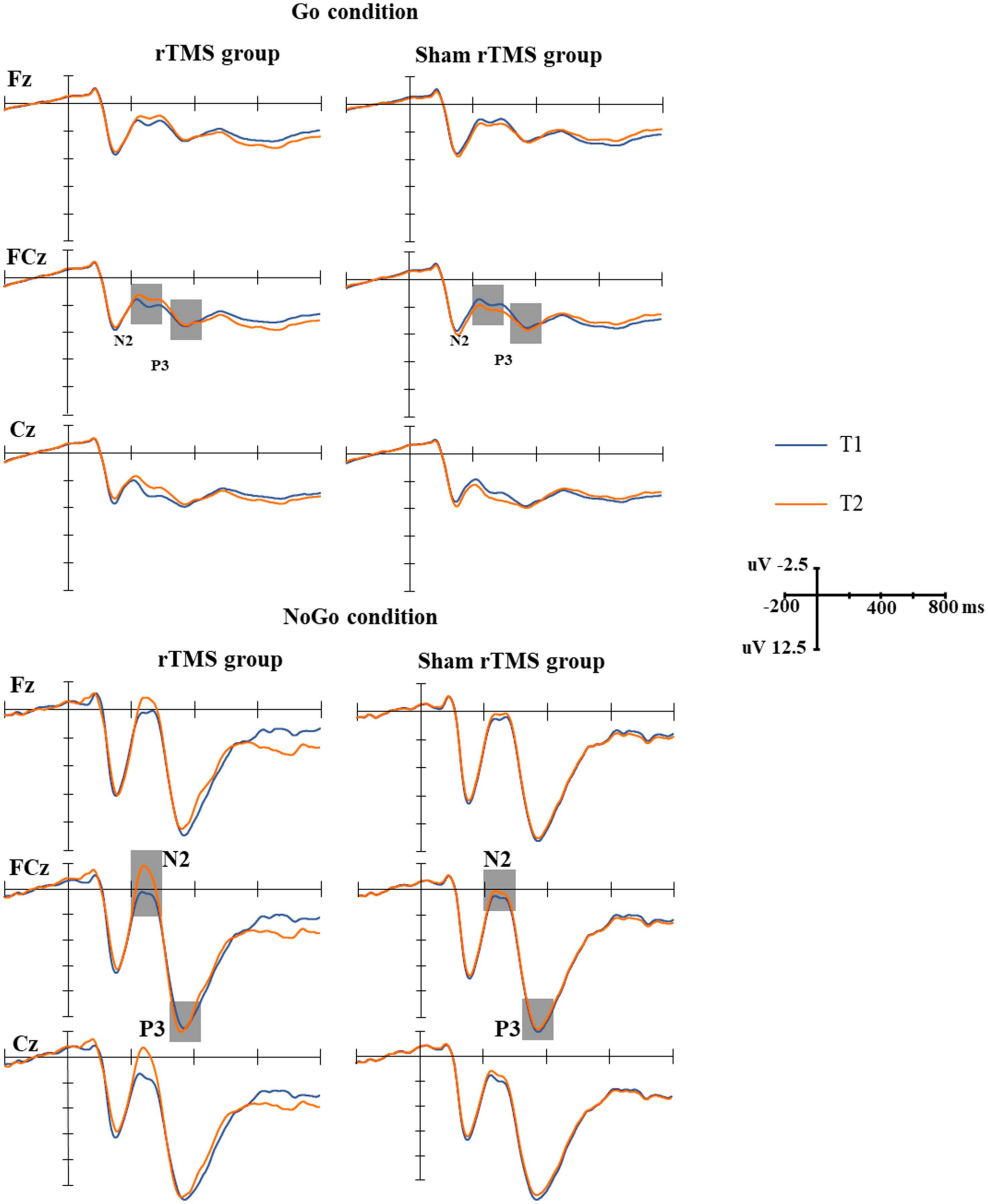
The grand-average N2 and P3 waveforms in the two groups. Upper: the grand-average N2 and P3 waveforms under Go conditions at two time points (T1, T2) in the rTMS group and sham rTMS group. The gray areas represent the time windows of the measured mean amplitudes of N2 (200–330 ms) and P3 (330–400 ms). Lower: the grand-average N2 and P3 waveforms under NoGo conditions at two time points (T1, T2) in the rTMS group and the sham rTMS group. The gray areas represent the time windows of the measured mean amplitudes of N2 (200–330 ms) and P3 (330–400 ms). T1: before stimulation; T2: immediately after 7 days of rTMS or sham rTMS.

The grand-average N2 waveforms in two conditions (Go, NoGo) at two time points in the rTMS group and the sham rTMS group are presented in Figure 3. The results revealed a significant main effect of time (*F*(1, 48) = 14.756, *p* = 0.001), i.e., the mean amplitude of the N2 component at T2 was more negative than that at T1 (T2: 3.815 uv, T1: 2.617 uv). The main effect of group was marginally significant (*F*(1, 48) = 3.042, *p* = 0.091). The N2 amplitude was larger in the rTMS group than in the sham rTMS group (rTMS group: 2.619 uv, sham rTMS group: 3.812 uv). A significant main type effect (*F*(1, 48) = 53.899, *p* < 0.001) was found, i.e., the mean amplitude of the N2 component was more negative in response to NoGo stimuli than in response to Go stimuli. An interaction effect of time × group (*F*(1, 48) = 17.533, *p* < 0.001) was found. Further analysis revealed that the mean amplitude of N2 in the rTMS group was larger than that in the sham rTMS group at T2 (*t* = −3.219, *p* = 0.003), whereas there was no significant difference with respect to this parameter at T1 (*t* = 0.156, *p* = 0.877). Additionally, a type × group interaction (*F*(1, 48) = 4.232, *p* = 0.048) was noted. Further analysis found that the mean amplitude of N2 in the rTMS group was larger than that in the sham rTMS group in the NoGo condition (*t* = −2.290, *p* = 0.029) but not in the Go condition (*t* = −0.051, *p* = 0.960). Most importantly, the time × type × group interaction was significant (*F*(1, 48) = 10.710, *p* = 0.003). Further analysis revealed that the mean amplitude of N2 for the NoGo condition was larger at T2 than at T1 in the rTMS group (*t* = 10.041, *p* < 0.001) but not for the Go condition (*t* = 0.465, *p* = 0.649), and there was no difference between T1 and T2 in the sham rTMS group for the Go condition or NoGo condition (Go condition: *t* = −0.100, *p* = 0.921; NoGo condition: *t* = −0.161, *p* = 0.874).

#### 3.2.2. P3 component

The grand-average P3 waveforms in two conditions (Go, NoGo) at two time points in the rTMS group and the sham rTMS group are presented in Figure 3. The results revealed a significant main effect of type of stimuli (*F*(1, 48) = 220.860, *p* < 0.001), i.e., the mean amplitude of the P3 component was more positive in response to NoGo stimuli than in response to Go stimuli (14.056 μv vs. 4.621 μv). There were no main effects of time (*F*(1, 48) = 0.030, *p* = 0.865) and group (*F*(1, 48) = 0.093, *p* = 0.763) and other interactions.

### 3.3. ^1^H-MRS data

The MI/Cr ratio in the left DLPFC was decreased by multiple sessions of rTMS over the left DLPFC. There was no significant difference in the NAA/Cr and Cho/Cr ratios between the rTMS group and the sham rTMS group before and after magnetic stimulation or sham stimulation.

#### 3.3.1. The NAA/Cr ratio of the bilateral DLPFC

There was no main effect of time (*F*(1, 48) = 0.035, −*p* = 0.852, *ηp*2 = 0.001), location (*F*(1, 48) = 3.636, *p* = 0.063, *ηp*2 = 0.070) or group (*F*(1, 48) = 0.005, *p* = 0.944, *ηp*2 = 0.000). No interaction effects of time × group (*F*(1, 48) = 0.134, *p* = 0.2716, *ηp*2 = 0.003), location × group (*F*(1, 48) = 0.376, *p* = 0.543, *ηp*2 = 0.008) or time × location × group (*F*(1, 48) = 0.854, *p* = 0.360, *ηp*2 = 0.017) were found. For the NAA/Cr ratio, there was no difference between the rTMS group and sham rTMS group before and after magnetic stimulation or sham stimulation.

#### 3.3.2. The Cho/Cr ratio of the bilateral DLPFC

Repeated measures analysis of variance showed that there was no main effect of time (*F*(1, 48) = 0.021, *p* = 0.885, *ηp*2 = 0.000), location (*F*(1, 48) = 3.532, *p* = 0.066, *ηp*2 = 0.069) or group (*F*(1, 48) = 0.775, *p* = 0.383, *ηp*2 = 0.016). No interaction effects of time × group (*F*(1, 48) = 0.029, *p* = 0.866, *ηp*2 = 0.001), location × group (*F*(1, 48) = 0.663, *p* = 0.420, *ηp*2 = 0.014) or time × location × group (*F*(1, 48) = 1.580, *p* = 0.215, *ηp*2 = 0.032) were found. In conclusion, for the Cho/Cr ratio, there was no difference between the rTMS group and the sham rTMS group before and after magnetic stimulation or sham stimulation.

#### 3.3.3. The MI/Cr ratio of the bilateral DLPFC

**Figure 4:**
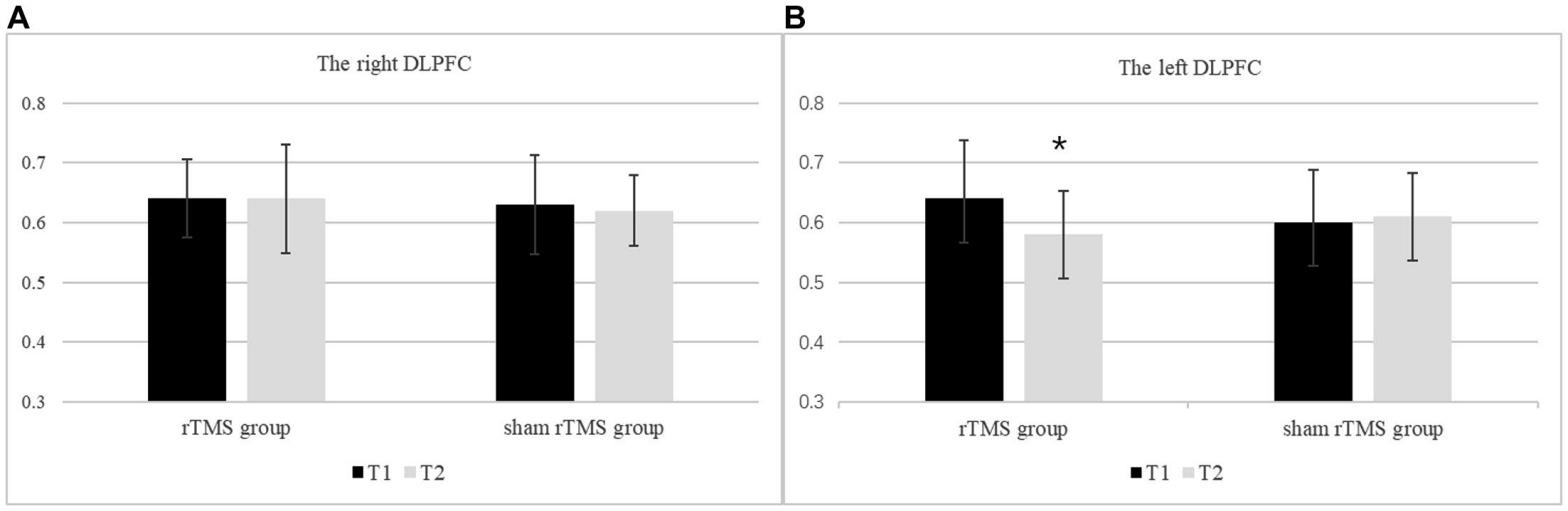
The MI/Cr ratio of the bilateral DLPFC at two time points in the rTMS group and the sham rTMS group. **(A)** The right DLPFC. **(B)** The left DLPFC. T1: before stimulation; T2: immediately after 7 days of rTMS or sham rTMS; * rTMS group T2 vs. T1: *p* < 0.05.

The MI/Cr ratio of the bilateral DLPFC at two time points in the rTMS group and the sham rTMS group are presented in Figure 4. The results revealed a significant main effect of group (*F*(1, 48) = 7.153, *p* = 0.010, *ηp*2 = 0.130), i.e., the MI/Cr ratio of the bilateral DLPFC was decreased in the rTMS group but not in the sham rTMS group. There was no main effect of time (*F*(1, 48) = 0.322, *p* = 0.573, *ηp*2 = 0.007) or location (*F*(1, 48) = 0.696, *p* = 0.408, *ηp*2 = 0.014). There were no interaction effects of time × group (*F*(1, 48) = 0.953, *p* = 0.334, *ηp*2 = 0.019), location × group (*F*(1, 48) = 0. 049, *p* = 0.826, *ηp*2 = 0.001) or time × location (*F*(1, 48) = 1.580, *p* = 0.215 *ηp*2 = 0.032). Further analysis revealed that the MI/Cr ratio of the left DLPFC decreased after rTMS (*p* = 0.036), but there was no significant change in the sham rTMS group.

## 4. Discussion

The present study focused on the effects and mechanism of multiple sessions of high-frequency rTMS (10 Hz) applied over the left DLPFC on response inhibition control in healthy young participants. The main results can be summarized as follows. There was a strong trend of decreasing commission errors of NoGo stimuli behaviorally and the amplitude of NoGo-N2 was significantly increased by high frequency rTMS over the left DLPFC. The differential effect was maximal at the FCz and Cz sites. That is, the success rate of inhibition for NoGo stimulation tends to increase after 10 Hz rTMS of the left DLPFC for 7 consecutive days. There were no effects on the RTs of correct Go trials, response accuracy for the Go condition or the amplitudes of Go-N2, Go-P3, and NoGo-P3. The MI/Cr ratio in the left DLPFC was decreased. However, there was no significant difference in NAA/Cr and Cho/Cr ratio between the rTMS group and the sham rTMS group before and after magnetic stimulation or sham stimulation.

The Go/NoGo paradigm used in the present study successfully replicated the NoGo effect; that is, NoGo-N2, and NoGo-P3 were significantly larger than Go-N2 and Go-P3 (Johnstone et al., 2007; Megías et al., 2017; Barry et al., 2018). This result suggested that our Go/NoGo paradigm was reliable. However, different from the present study, many previous studies have found no effects. For example, Upton et al. (2010) found that low-frequency rTMS (1 Hz) over neither the left DLPFC nor the right DLPFC affected the behavioral results and the ERP waveforms of the signal stop paradigm. Similarly, Grossheinrich et al. (2013) did not obtain any behavioral effect in a Go/NoGo task after stimulating the left DLPFC with 1 Hz rTMS. Huang et al. (2004) also found that short-term 5 Hz rTMS over the left DLPFC could neither improve nor worsen the behavioral performance of the Go/NoGo task in a crossover experiment. The reasons why the above studies did not obtain positive results may be as follows. (1) The sample sizes of the three studies were 14 cases, 24 cases and 10 cases, which are relatively small. (2) Single-session or short-term rTMS was adopted. (3) Low-frequency (less than 5 Hz) rTMS was used in the above studies. (4) It is difficult to solve the problems of the long-term effect of rTMS in the crossover experimental designed study. To avoid the limitations noted above, a randomized, sham stimulation controlled experimental design with a medium sample size (25 cases in each group) and multiple sessions of high-frequency rTMS was adopted in the present study.

The results showed that there was a strong trend of decreasing commission errors of NoGo stimuli behaviorally by high-frequency rTMS over the left DLPFC; that is, after 10 Hz rTMS for 7 consecutive days, the success rate of inhibition for NoGo stimuli tends to increase, and the RTs of Go stimuli was not prolonged. We suggest that multiple sessions of high-frequency rTMS over the left DLPFC has significant underlying value to improve the response inhibition control of young healthy participants and improve behavioral performance in a Go/NoGo task. From the perspective of electrophysiology, multiple sessions of high-frequency rTMS significantly increased the amplitude of NoGo-N2 and had no effect on Go-N2. According to previous studies, NoGo-N2 is an enhanced negativity at approximately 200–400 ms poststimulus onset in response to NoGo stimuli and may reflect the early stage in the time course of the dynamic processing of response inhibition control, such as conflict monitoring or detection, i.e., the conflict between the internal representation of the Go response and the NoGo stimulus (Megías et al., 2017; Kok, 1986; Smith et al., 2008; Wu et al., 2010). For the P3 component, the average amplitude of NoGo-P3 at two time points in the two groups was not affected by rTMS. Different from our initial experimental expectation, multiple sessions of high-frequency rTMS significantly increased NoGo-N2 but did not significantly reduce the amplitude of NoGo-P3. It is mentioned above that NoGo-P3 reflects a later stage of the response inhibitory process, which represents the underpinning of the action outcome assessment (Smith et al., 2008). The present study supports the functional separation theory of NoGo-N2 and NoGo-P3 (Bokura et al., 2005; Moyle et al., 2006). That is, the multiple sessions of high-frequency rTMS over the left DLPFC affected the early stage instead of the later stage in the time course of the dynamic processing of the response inhibition control and increased the amplitude of NoGo-N2. Based on the physical characteristics of rTMS, the results we obtained are reasonable. It is generally believed that low-frequency (<1 Hz) rTMS is likely to cause inhibition of neuron, whereas high-frequency (5–25 Hz) rTMS inversely leads to neuronal depolarization and excitation under the stimulating coil in a localized area (Haraldsson et al., 2004). The component of NoGo-N2 was affected by rTMS. It has shown that low frequency (1 Hz) rTMS over the left DLPFC decreased the amplitude of NoGo-N2 according to previous literature (Grossheinrich et al., 2013). In the present study, the amplitude of NoGo-N2 was increased after high-frequency rTMS. The amplitude of NoGo-N2 represents the amount of cognitive resources involved in the early stage of response inhibition control. The mean amplitudes of NoGo-N2 and NoGo-P3 in PD patients were significantly lower than those in healthy controls (Bokura et al., 2005). In the present study, rTMS improved the response inhibition control of young healthy participants from the perspective of electrophysiology. The DLPFC-ACC circuit is an important neural structure in response inhibition control despite a large and complex neural network is involved. Therefore, the increase in NoGo-N2 may be related to the increase in excitability of the neural circuit.

The biological mechanisms underlying the effects of rTMS on neuropsychiatric disorders and cognition are complicated and remain largely unclear. The metabolic changes in local brain regions may be one of the biological mechanisms underlying the effects of rTMS on neuropsychiatric disorders and cognition (Baeken et al., 2015). The contents of NAA and Cho in the ACC were significantly lower in patients with depression than in normal individuals (Zheng et al., 2015). After high-frequency rTMS treatment over the left DLPFC for 4 weeks (15 Hz, 110% stimulation intensity, 20 sequences), the NAA content in the ACC increased significantly in patients with depression. Another study obtained similar results that rTMS can improve the performance of the Hopkins oral learning test and short visual spatial memory test, increase the ratio of NAA/Cr and Cho/Cr, and make it close to normal (Qiao et al., 2016). As mentioned above, NAA is one of the markers of neurons. Changes in NAA concentration can reflect the activity of neurons in different disease states, such as major depression and Alzheimer’s disease. A decrease in NAA concentration indicates the loss of neurons or the loss of neuronal function.

In the present study, we found that rTMS can potentially improve behavioral performance and increase the amplitude of NoGo-N2 in a Go/NoGo task and thus improve response inhibition control. It is speculated that multiple sessions of high-frequency rTMS over the left DLPFC can not only recruit more neural resources but also enhance the efficiency of resources to solve cognitive problems. If the NAA concentration is confirmed to be increased after rTMS, the result will be perfect. However, the results showed that high frequency rTMS did not increase the NAA concentration in the left DLPFC with ^1^H-MRS. The possible reasons are as follows: (1) the method of ^1^H-MRS is still not sensitive enough. Detecting metabolism in living tissue using ^1^H-MRS is easily disturbed by water, skull and lipid signals. The location of the bilateral DLPFC is close to the skull, and the results may be affected, although the interference of these signals can be controlled to the greatest extent. (2) Ceiling effect (Mir-Moghtadaei et al., 2015): rTMS can increase the NAA concentration in the ACC of patients with depression, which is significantly lower than that in healthy people. However, the participants selected in the present study were healthy young people whose brain structure and metabolism were normal. The NAA concentration was not increased further after rTMS.

Interestingly, we found that the MI/Cr ratio of the left DLPFC decreased after multiple sessions of high-frequency rTMS over the left DLPFC. MI is a precursor of phosphatidylinositol and 4,5-diphosphate phosphatidylinositol that participates in the transmission of intracellular information and is also an important osmotic regulator (Haris et al., 2011). The MI/Cr ratio increases in patients with many neuropsychiatric diseases, such as bipolar disorder, because abnormal phosphatidylinositol levels are one of the pathological bases of many neuropsychiatric diseases. Increased MI was found in the prefrontal lobe, temporal lobe, cingulate gyrus, and basal ganglia in patients with bipolar disorder, while lithium carbonate treatment can reduce the level of phosphatidylinositol (Silverstone et al., 2005). Similar results were found in patients with Alzheimer’s disease. The MI concentration and MI/Cr ratio are increased in patients with Alzheimer’s disease (Miller et al., 1993; Huang et al., 1999; Rose et al., 1999) and may be related to the occurrence of amyloid deposition and neurofibrillary tangles in the brains of patients with Alzheimer’s disease. In addition, the MI/Cr ratio of the bilateral DLPFC increased in migraine patients with depression (Lirng et al., 2015). The occurrence of depressive symptoms in migraine patients is thought to be related to an increase in the MI/Cr ratio. Although it is still uncertain whether the increase in the MI/Cr ratio is the ultimate cause or secondary to the changes in other substances in the pathological state, the increase in the MI/Cr ratio indicates the abnormal transmission of the second messenger signal. In the present study, rTMS reduced the MI/Cr ratio of the left DLPFC, which may be one of the metabolic bases of the positive effect of rTMS. Further animal experiments are needed to confirm this assumption in the future.

The current study has certain limitations that must be addressed. First, as mentioned above, longer stimulation trains and longer treatment durations would induce more lasting effects (Eisenegger et al., 2008). In other words, more pulses, higher intensity and longer treatment durations mean better effects to some extent with the control of safety and adverse effects of rTMS. Therefore, more exposure to rTMS may be needed in the future studies. Second, the sample size might still not large enough. The generalizability of the current findings may also be limited by our utilization of a relatively small sample of young adults. Therefore, additional studies with larger samples are warranted. Third, in our study, the conventional “5 cm rule” (the method of Pascual-Leone) was applied to target the left DLPFC. This conventional method is not entirely accurate compared with MRI-guided neuronavigation, which can target a desired cortical region directly (Mir-Moghtadaei et al., 2015). However, many previous studies have used this technique, and the outcomes have been robust and reproducible (Haghighi et al., 2015). Fourth, our study focused on the cumulative effects of rTMS; however, the long-term effects and the duration of the positive effects of rTMS are unclear.

## 5. Conclusion

In conclusion, the results of the current study suggest that multiple sessions of high-frequency rTMS over the left DLPFC can potentially decrease the commission errors in the NoGo condition and increase the amplitude of NoGo-N2 significantly. The MI/Cr ratio in the left DLPFC was decreased by multiple sessions of rTMS over the left DLPFC. This observation supports the view that high-frequency rTMS over the left DLPFC improves the response inhibition control of healthy young participants. We suggest that the increase in NoGo-N2 amplitude may be related to the increased excitability of the DLPFC-ACC neural loop. Metabolic changes in the left DLPFC may be a possible mechanism for the improvement of cognition by rTMS. Further research is required to expand rTMS applications by involving more diverse groups of participants and investigating the long-term effects of rTMS.

## Data availability statement

The raw data supporting the conclusions of this article will be made available by the authors, without undue reservation.

## Ethics statement

The studies involving human participants were reviewed and approved by The Ethics Committee of the First Hospital of Hebei Medical University. The patients/participants provided their written informed consent to participate in this study.

## Author contributions

YL and MW: project administration. YL, JW, and WW: data curation. YL, QB, LL, and XW: formal analysis and methodology. YL: funding acquisition and resources. YL and JP: investigation, writing—original draft, and writing—review and editing. YL and WW: software. MW: supervision. All authors contributed to the article and approved the submitted version.

## Funding

Projects of key research and development of Hebei Province, 172777221 The Spark Program, XH201705.

## Conflict of interest

The authors declare that the research was conducted in the absence of any commercial or financial relationships that could be construed as a potential conflict of interest.

## Publisher’s note

All claims expressed in this article are solely those of the authors and do not necessarily represent those of their affiliated organizations, or those of the publisher, the editors and the reviewers. Any product that may be evaluated in this article, or claim that may be made by its manufacturer, is not guaranteed or endorsed by the publisher.
